# Supplementary material for: Surfactants Screen Slide Electrification
Source: Angew Chem Int Ed Engl. 2025 Jul 1;64(31):e202423474. doi: 10.1002/anie.202423474 (PMC12304857; doi:10.1002/anie.202423474)
Supplement: Supplementary file 1 — Supporting Information [file ANIE-64-e202423474-s001.docx]

**Supporting information for**

**Surfactants Screen Slide Electrification**

**SI 1. Preparation of surfactant solutions and surfaces**

Substrate’s cleaning and preparation: (1) quartz substrates (76.2×25.4×1.0 mm^3^, Thermo Fisher Scientific) were cleaned in an ultrasonic bath in ethanol (absolute, VWR Chemical) for 10 min. After drying by N_2_ blowing, we further cleaned them with O_2_-plasma at 300 W for 10 min (Femto low-pressure plasma system, Diener electronic). (2) Gold substrates were prepared by sputter coating (BalTec MED 020), with 5 nm chromium and 35 nm gold on glass slides (76.2×25.4×1.0 mm^3^, Paul Marienfeld). The glass slides were cleaned in the same way as quartz substrates before use. After sputter coating, gold substrates were used immediately without further cleaning.

Surface preparation: (1) Teflon-gold and Teflon-quartz were prepared by dip coating with 1(wt)% Teflon AF1600 (Sigma-Aldrich) in FC-75 (97%, Fisher Scientific). The dip speed is 90 mm/min while the pull speed is 10 mm/min. After drop coating, the samples were vacuum-annealed for 24 hours at 160 $℃$ in the oven before use. The thickness of the Teflon film was around 60 nm measured by a profiler (P-7 stylus profiler, KLA-Tencor). (2) PS-quartz was prepared with 1(wt)% PS ($M_{w}=192 \mathrm{kg}\mathrm{mol}^{-1}$, Sigma-Aldrich) in toluene (99.8%, Sigma-Aldrich). Both the dip speed and pull speed used are 90 mm/min. After drop coating, the samples were vacuum-annealed for 24 hours at 120 °C before use. (3) PFOTS-quartz was prepared via chemical vapor deposition in a vacuum desiccator (< 100 mbar.) with 0.5 ml of 1H, 1H, 2H, and 2H-perfluorooctadecyltrichlorosilane (97%, Sigma-Aldrich) for 30 min. Before use, we rinsed the samples with ethanol to get rid of unbounded silanes. (4) PDMS-quartz was prepared by the “grafting to” method with a few drops of silicone oil ($M_{w}=6 \mathrm{kg}\mathrm{mol}^{-1}$; Alfa Aesar) spread on the quartz substrates for 24 hours at room temperature. Before use, we cleaned the sample with ultrasound in toluene, ethanol, and distilled water for 10 min each to remove the unbound silicone oil. (5) Soot-templated superhydrophobic surfaces were prepared followed the same method reported by Deng *et al.* ^[45]^. In short, a soot layer first coated by holding a glass slide above the flame of a paraffin candle. To make the soot coting homogenous, we moved the glass slide forward and back on top of the flame for 1 min. Then a silica shell was coated on the soot particles using tetraethoxysilane (Sigma-Aldrich, 98%) and an aqueous ammonia solution (TCI, 28%) via chemical vapor deposition (CVD) at ambient temperature for 24 h. Then the samples were calcined at 600°C for 2 h in an oven. After they cool down to the room temperature, we activated the samples with O_2_ plasma treatment for 10 min and then fluorinated using trichloro(1H,1H,2H,2H-perfluorooctyl)silane via CVD for 30 min.

**SI 2. Charge measurement of sliding drops**

The samples were mounted on a grounded tilting stage in a grounded Faraday cage with a tilted angle of 50$^{\circ}$ for PFOTS-quartz and PDMS-quartz samples, 40° for PS-quartz, and 25° for Teflon-quartz. The tilted angles were adjusted for different samples to ensure the drop speed is similar and to avoid drop pearling. Drops were deposited onto the samples with a grounded syringe needle (outer diameter: 2 mm) connected to a peristaltic pump (Gilson Minipuls 3, Wisconson, USA) with a drop interval of 15 s. The size of syringe needle is fixed for different solutions. Thus, the drop volume changes with surfactant concentration from around 45$\mu L$ to 2$5 \mu L$ for CTAB and SDS while from around 45$\mu L$ to 16$\mu L$ for C_8_E_3_. The sliding drops first touch a grounded electrode for charge neutralization. After sliding for ~4 cm, the sliding drops touch a second electrode connected to a low noise current amplifier (FEMTO DDPCA-300, Berlin, Germany) for current detection. The DDPCA-300 produced by Femto is a low noise transimpedance amplifier with a gain from 10^4^ to 10^13^ V/A and a bandwidth of 400 Hz. The circuit is available under <https://www.femto.de/images/pdf-dokumente/de-ddpca-300.pdf>. We recorded the data with a National Instruments data acquisition card (NI USB-6366 X-Series) and the accompanying LabVIEW software. During drop sliding, an Ionizing Air Blower (Simco-Ion, Aerostat PC Ionizing Air Blower, USA) was running in front of the samples, to ensure every drop sliding on neutral surfaces. To avoid surfactant residue affecting the charge measurement by the metal electrode, it was rinsed by water before each measurement. The drop charge is calculated by integrating the peak current with the corresponding time, a typical time value is $\sim$2 ms for water drop as showed in the inset of figure 1b. The uncertainty of drop charge is determined by the standard deviation of measurements on three fresh samples.

**SI 3. Velocity and contact angle measurement of sliding drops**

We recorded sliding drops on tilted samples by a high-speed camera (1000 fps, FASTCAM Mini, UX100 Photron) from side by a telecentric lens (TitanTL telecentric lens, $\times$0.268, C-mount, Edmund Optics) with a resolution of $\sim$37 μm per pixel. Before each measurement, the surfaces were neutralized by ionic air blowing for 15 min. During video recording for subsequence drops with a drop interval of 15 s, we turned off the ionic air blowing to avoid its influence on drop motion. The velocity and contact angles of sliding drops were analyzed by 4-segment super-resolution optimized-fitting toolkit ^[46]^. The velocity is calculated based on the position of the center of the drop. The uncertainty of the analysis is mainly from the determination of the drop contour with semi-pixel accuracy, which normally results in a very low error (≪1%) in drop velocity even with a displacement in baseline determination and title angle ^[46]^.

**SI 4. Zeta potential measurement**

Zeta potential was measured by an electrokinetic analyzer (SurPASS 3, Anton Parr, Austria). For each solution and each kind of surfaces, we measured two different samples at 3 different positions for each sample. We took the average zeta potential measured at 6 positions and their standard deviation as errors for the plotting.

**SI 5. Fluorescence image scanned by the laser scanning confocal microscopy (LSCM)**

Water drops are dissolved with the fluorescent molecules at a concentration of 0.1 mM for fluorescein (free acid, 95%, Sigma-Aldrich, CAS#2321-07-5, the neutral fluorescein can partially dissociate into anionic version at pH<7), rhodamine 6G (99%, Sigma-Aldrich), and eosin G (75%, Sigma-Aldrich). A drop, with a volume of 35±5 µL, slides down the 50° tilted surface on the tilted plate setup (Fig.1a). The drop starts sliding without any initial charge by being neutralized with a 0.025-mm-diameter grounded tungsten wire, right after they land on the surface. The sliding motion persisted for 4-5 cm. Experiments were carried out at a temperature of 22±2°C and a relative humidity of 35±5%. LSCM images were taken using an inverted confocal microscope (Leica TCS SP8 SMD) with the Leica LAS X software. Horizontal visualization of the molecules adsorbed on surfaces is obtained by a HC PL FLUOTAR 2.5$\times$/0.07 CORR CS DRY objective. The scan speed is 400 Hz and scan mode is xyz scanning. For observation of molecules from Rhodamine 6G, Eosin Y, and Fluorescein on PS, PFOTS and Teflon surfaces, an argon laser line (514 nm, 10 -15 mW) was used for excitation. The excitation light intensity can be controlled by an Acousto-optical tunable filter (AOTF), whose transmission was set to 80%. The collected emission was detected in the wavelength range between 535 nm and 580 nm with a detector gain value set to 800/1250. Notably, for molecules absorbed on PDMS surfaces, the gain value was adjusted to 1250/1250 to enhance signal clarity. Confocal images were transformed into a data matrix of 6.2 $\times$ 6.2 mm^2^ dimensions (pixel size: 0.012 $\times$ 0.012 mm^2^) and subsequently amalgamated to delineate the trajectory of adsorbed molecules.

**SI 6. Charge measurement of bouncing drops**

The tested samples were placed on a PTFE plate with 1 mm-depth groves. Two copper plates of 50$\times$16$\times$4 mm^3^ with a gap of 2.5 mm were placed vertically inside of groves. We applied a voltage of 3 KV by a high voltage DC power supply (Analogic, AN 3200) between the two cooper plates during measurements. Drops were released from a syringe (100 µL, Model 710 N SYR, Hamilton) from around 2.5 cm above the surfaces. To avoid drop charging due to polarization effects by the electric field, a grounded aluminum tube (20 mm height, 15 mm outside diameter, 2 mm inner diameter) shielded the drops during detachment from the needle. We recording drop bouncing from side with the same a high-speed camera but different telecentric lens (1.0$\times$ SilverTL, 10 μm per pixel, Edmund Optics) as above for sliding drop.

**SI 7. Analytical and numerical models**

1. **Analytical model**

We model the surface chemistry with a surface charge regulation model includes two reactions, one for negative and one for positive charging of the surface. The general reaction equations read ^[14]^:

$R-OH\overset{K_{A}}{\overbrace{\leftrightarrow}}R-O^{-}+H^{+}$,

$H^{+}+R-OH\overset{K_{B}}{\overbrace{\leftrightarrow}}R-OH_{2}^{+}.$ (S1)

We assume 1) a fixed number of indistinguishable, amphoretic surface sites $R-OH$, each can bind one charge; 2) a symmetric electrolyte; 3) the reaction is always in equilibrium. According to the law of mass action, equilibrium constants $K_{\text{A}}$ and $K_{\text{B}}$ are:

$K_{\text{A}}=\frac{\alpha c_{+}}{\left( 1-\alpha-\beta\right)}$ and $K_{\text{B}}=\frac{\beta}{\left( 1-\alpha-\beta\right)c_{+}}$. (S2)

With the occupancy rates for positively and negatively charged sites $\alpha:=\frac{\left[ \text{R-}\text{O}^{\text{-}} \right]}{\Gamma}$ and $\beta:=\frac{\left[ \text{R-O}\text{H}_{\text{2}}^{\text{+}} \right]}{\Gamma}$, respectively. The brackets indicate surface concentrations and $\Gamma$ is the concentration of surface sites. Since the deposited surface charge density $\sigma_{s}$ is the difference in charge density between the oppositely charged surface sites, it can be expressed in terms of the equilibrium constants and the proton concentration $c_{+}$, which is the local proton concentration at the solid-liquid interface:

$\sigma_{s}=-\Gamma ze\left( \alpha-\beta\right)=\Gamma ze\frac{K_{\text{B}}c_{+}^{2}-K_{\text{A}}}{K_{\text{B}}c_{+}^{2}+c_{+}+K_{\text{A}}}$. (S3)

In which, the local proton concentration is determined by Boltzmann equation:

$c_{+}=c_{+,0}\cdot\exp\left( \tilde{\zeta} \right)$. (S4)

The equilibrium constants determine the point of zero charge and the zeta potential, so that the equilibrium constants at a point of zero charge is $3.55$ and a zeta potential is $-55 \mathrm{mV}$ with a Debye length of $100 \mathrm{nm}$ and surface site density of $5 \mathrm{nm}$ for water without surfactant ^[18]^.

In the presence of anionic surfactant, the anionic surfactant adsorbs to the substrate due to the hydrophobic force, which results in an increase in negative zeta potential ($\tilde{\zeta}$) (Figure 2). According to equation (S4), the increase in the zeta potential caused by the anionic surfactant changes the local ion concentration ($c_{+}$) and thus shifts the equilibrium towards a weaker bound surface charge according to equation (S3). We assume 1) the adsorbed surfactant does not reduce the surface site density ($\Gamma$is constant) but acts as an additional source of surface charge besides primary ions, so that the total surface charge in the electric double layer is $\sigma_{\mathrm{eq}}=\sigma_{\mathrm{primary}}+\sigma_{\mathrm{surfactant}}$; 2) the surface charge after dewetting is only due to the binding of primary ions since anionic surfactant desorbed again during dewetting; 3) the bulk proton concentration is kept constant. According to equation (S3 and S4), we calculated the bound surface charge which quantifies the maximum possible surface charge deposited after dewetting as a function of zeta potential. The result shows that an increase in effective zeta potential from -35 mV to -70 mV leads to a decrease in the bound surface charge from 75 mC/m^2^ to 14 mC/m^2^ (Figure S6). Thus, the presence of anionic surfactant as additional source of surface charge in the electric double layer reduces the binding of primary ions which contributes to the charge deposition after dewetting.

1. **Numerical model**

In the presence of nonionic surfactant, the zeta potential didn’t change much (Figure 2a). As the hydrophobic tail of the surfactant does not allow to form hydrogen bonds with neighboring water molecules, an entropic force, known as hydrophobic force, is created which drives the surfactant towards the substrate where it is absorbed. The surfactant thus covers a part of the surface and we expect that the surfactant reduces the available surface sites to which charges can be bound. Since Equation (S3) suggests a linear relationship between surface site density and surface charge, we investigate the effect of reducing the surface site density on the surface charge by numerical simulation in this section.

Simulations are performed using the finite-element code Comsol Multiphysics, Version 6.1. The computational domain is a two-dimensional wedge with an opening angle $\theta$ and a radius of $L=100\lambda_{\text{DI}}=96 \text{μm}$ ($\lambda$: Debye length, subscript: DI water) representing the idealized dewetting tail of the drop. The solid surface is assumed to be perfectly clean, flat, and smooth.

Figure S6 shows the computational domain and the grid structure. To eliminate finite-size effects, we expand the computational domain by a predomain with a length of $L_{\text{pre}}=c_{\text{pre}}L$. The computational domain is discretized with a structured grid consisting of quadrilateral elements, which are refined towards the interfaces and the contact line based on a geometric series. Additionally, a finely resolved boundary layer of cells with a thickness of $\lambda$ is defined at the solid-liquid interface. The velocity is discretized with quadratic and all other variables with linear shape functions. The relative error due to grid and finite size are both below 0.005, The relative error of the numerical solver was set to 0.001. Grid convergence and finite size effects was verified by Ratschow *et al.* ^[18]^ and yield a predomain length of $c_{pre}=4$. We consider a frame-of-reference co-moving with the contact line.

The mathematical model is governed by Stokes and Poisson-Nernst-Planck (PNP) equations for an incompressible, Newtonian liquid, which are coupled by the space charge density ($\rho_{v}$). The electric body force is neglected as its influence is below the numerical accuracy. At the gas-liquid interface [I], we prevent flow and species flux across the boundary. Furthermore, we account for the low viscosity and relative permittivity of gas in comparison to aqueous solutions and apply a vanishing shear stress and a vanishing normal electric field. Along the solid surface [II], the moving contact line is modeled by a tangential wall velocity which equals the drop velocity, and the Navier Slip boundary condition. Normal flow across the solid-liquid interface is set to zero. However, individual species may have a non-zero flux due to adsorption and dissociation processes which is captured by a charge regulation model including two reaction equations. Additionally, bound surface charge moves along the solid surface relative to the frame-of-reference co-moving with the contact line. Consequently, we complement the boundary conditions by a surface charge conservation equation which balances the movement of surface charge along the solid-liquid interface with charge transfer from the liquid. At the boundary to the bulk liquid [III], an isobaric boundary condition is applied. Additionally, we approximate the potential and species concentrations for the PNP equations by the solution of the Poisson-Boltzmann equation for an infinite flat plate. All governing equations and the corresponding boundary conditions are given in table S2. The parameters used are given in table S3. For any further information on the simulation, the reader is referred to ^[18]^.

In the simulation, the surface site density is systematically reduced under the assumption that the surfactant does not affect the zeta potential and the Debye length. Figure S8 shows the scaled surface charge at the contact line over the surface site density normalised to the standard surface site density of $55 \mathrm{nm}^{-2}$. The surface charge at the contact line determines the deposited surface charge after dewetting. The simulation is performed for three different Péclet numbers $Pe=U \lambda/D_{+}$. With decreasing surface site density, the amount of surface charge decreases. However, according to equation (S4), this also leads to a change in the local ion concentration, which counteracts the effect. Thus, the surface chemistry has a buffering effect that dampens the effect of surface site reduction. This effect is even less significant at higher Péclet numbers. Thus, the reduction of surface charge deposition due to the occupy of active surface sites by non-ionic surfactant is not so significantly.

**SI 8. Estimation of the deposited dye amount after dewetting**

To estimate an upper limit of the surface density of deposited Rhodamine 6G, we considered the reduction in drop charge. The charge of a pure water drop was +0.62 (0.43) nC after sliding 4 cm on a Teflon (PS) surface. With 0.1 mM dye, the drop charge was only +0.05 (-0.16) nC after sliding the same distance (Figure 2c). The difference is Δ*Q* = 0.57 (0.56) nC. Assuming that the same number of primary ions are deposited and the difference is due to co-deposited dye, the number of deposited Rhodamine 6G can be calculated as ^[47]^: $\text{n=Δ}\text{Q}/{\text{e}\text{Z}}$. Here, *e* is the elementary charge and *Z* is the valency of one dye molecule at neutral pH. Taking *Z* = 1 and assuming that the dye molecules are evenly distributed over a drop path area of say 40 $\times$ 4 mm^2^ this would lead to about 2.2$\times$10^13^ molecules/m^2^. This corresponds to a mean spacing between deposited rhodamine 6G molecules of $\approx$200 nm.

**Table S1.** Static contact angles of water drops with different surfactant concentrations on the Teflon-gold surfaces. The error is $\pm2^{\circ}$ calculated by the standard deviation of three measurements.

| Liquid | Water | **10% CMC surfactant in water** | | | **100% CMC surfactant in water** | | |
| --- | --- | --- | --- | --- | --- | --- | --- |
|  |  | CTAB | SDS | C_8_E_3_ | CTAB | SDS | C_8_E_3_ |
| Contact angle ($^{\circ}$) | 115 | 110 | 112 | 101 | 95 | 93 | 74 |

**Table S2**. Governing equations and boundary conditions for the numerical simulations. Here, $\boldsymbol{n}$ and $\boldsymbol{t}$ denote outward normal and tangential vectors in a right-handed system, and the coordinate $y$ measures the distance to the solid-liquid interface [III]. The symbol for the pressure is $p$, for the velocity field $u$, for the electric potential $\Psi$, for the space charge density $\rho_{v}$, for the species flux $\boldsymbol{J}_{\boldsymbol{i}}$, for the species concentration $c_{i}$, for the bulk species concentrations $c_{i,0}$, for the diffusivity $D_{i}$ and for the species valence $z_{i}$. The subscripts $i=+$ and $i=-$ represent cations and anions, respectively. Additionally, $\eta$ represents the dynamic viscosity, $\varepsilon=\varepsilon_{\text{0}}\varepsilon_{\text{l}}$ the permittivity, $T$ the temperature, $R$ the gas constant, $e$ the elementary charge and $F$ the Faraday constant. The surface divergence operator is $\boldsymbol{\nabla}_{\text{s}}=\boldsymbol{\nabla}-\boldsymbol{n}\cdot\left( \boldsymbol{n}\cdot\boldsymbol{\nabla} \right)$. Additional quantities and parameters are the shear stress $\boldsymbol{\tau}=\eta\left[ \boldsymbol{\nabla}\boldsymbol{u}+\left( \boldsymbol{\nabla}\boldsymbol{u} \right)^{T} \right]$, the wall velocity $u_{w}$ and the drop velocity $U$. The slip length is denoted as $l_{\text{s}}$, the surface site density is $\Gamma$ and the Debye length $\lambda$. The reaction constants $K_{A}$ and $K_{B}$ to calculate the surface charge density $\sigma$ are calculated by the point of zero charge and the zeta potential. The thermal potential is $\phi_{T}=kT/e$ and the normalized zeta potential is $\tilde{\zeta}=\zeta/\phi_{T}$.

|  | **Stokes** | **Poisson-Nernst-Planck** |
| --- | --- | --- |
| Governing equations | $0=-\nabla p+\eta\boldsymbol{\nabla}^{2}\boldsymbol{u}$  $\boldsymbol{\nabla\cdot u}=0$ | $\boldsymbol{\nabla}^{2}\Psi=-\varepsilon^{-1}\rho_{v}$ with $\rho_{v}=F\sum z_{i}c_{i}$  $\boldsymbol{\nabla\cdot}\boldsymbol{J}_{\boldsymbol{i}}=0$  $\boldsymbol{J}_{\boldsymbol{i}}=c_{i}\boldsymbol{u}-D_{i}\boldsymbol{\nabla}c_{i}-z_{i}D_{i}\left( RT \right)^{-1}Fc_{i}\boldsymbol{\nabla}\Psi$ |
| [I] gas-liquid interface | $\boldsymbol{u}\boldsymbol{\cdot}\boldsymbol{n}=0$  $\boldsymbol{\tau\cdot}\boldsymbol{t}=0$ | $\boldsymbol{E}\boldsymbol{\cdot}\boldsymbol{n}=0$  $\boldsymbol{J}_{\boldsymbol{i}}\boldsymbol{\cdot}\boldsymbol{n}=0$ |
| [II] boundary to bulk liquid | $\boldsymbol{\tau}=\boldsymbol{0}$ | $\Psi=2z^{-1}\ln\left[ \frac{1+\exp\left( -\lambda^{-1}y \right)\tanh\left( \tilde{\zeta}/4 \right)}{1-\exp\left( -\lambda^{-1}y \right)\tanh\left( \tilde{\zeta}/4 \right)} \right]$  $c_{i} =c_{i,0} \exp\left( \frac{z \Psi}{\Phi_{T}} \right)$ |
| [III] solid-liquid interface | $\boldsymbol{u}\boldsymbol{\cdot}\boldsymbol{n}=0$  $u_{\mathrm{slip}}=\left( u_{w}-\boldsymbol{u} \right)\cdot\boldsymbol{t}$  $u_{\mathrm{slip}}=l_{s}\boldsymbol{\nabla}_{s}\cdot\boldsymbol{u}$, $u_{w}=U\boldsymbol{t}$ | $\sigma=-\Gamma ze\left( \alpha-\beta\right)=\Gamma ze\frac{K_{\text{B}}c_{+}^{2}-K_{\text{A}}}{K_{\text{B}}c_{+}^{2}+c_{+}+K_{\text{A}}}$  $\boldsymbol{E}\boldsymbol{\cdot}\boldsymbol{n}=-\varepsilon^{-1}\sigma$  $-U\boldsymbol{\nabla}_{s}\sigma_{s}=Fz_{+}\boldsymbol{n}\boldsymbol{\cdot}\boldsymbol{J}_{\boldsymbol{+}}$ and $\boldsymbol{n}\boldsymbol{\cdot}\boldsymbol{J}_{\boldsymbol{-}}=0$ |

**Table S3**. Reference conditions, electrolyte and surface properties for the simulation.

| **Parameter** | **Value** | **Description** | **Reference** |
| --- | --- | --- | --- |
| $T$ | $298.15 K$ | Temperature |  |
| $\eta\left( T=298.15 K \right)$ | $8.93\cdot{10}^{-4} Pas$ | Dynamic viscosity | COMSOL library |
| $\rho\left( T=298.15 K \right)$ | $998.21 \mathrm{kg}/m^{3}$ | Density | COMSOL library |
| $\varepsilon_{l}\left( T=298.15 K \right)$ | $78.3$ | Relative permittivity of fluid | [2] |
| $D_{-}$ | $9\cdot{10}^{-9}m^{2}s^{-1}$ | Diffusion coefficient of anions | [48] |
| $D_{+}$ | $9\cdot{10}^{-9}m^{2}s^{-1}$ | Diffusion coefficient of cations | [48] |
| $\lambda$ | $100 nm$ | Debye length | [2] |
| $l_{s}$ | $1 nm$ | Slip length | [49, 50] |
| $z_{+}$ | $1$ | Species’ valence |  |
| $K_{A}$ | $4.33\cdot{10}^{-4} m^{3}/mol$ | Equilibrium constant corresponding to positively charged surface groups |  |
| $K_{B}$ | $3.44\cdot{10}^{-5}\mathrm{mol}/m^{3}$ | Equilibrium constant corresponding to negatively charged surface groups |  |
| $\theta$ | $80^{\circ}$ | Contact angle |  |


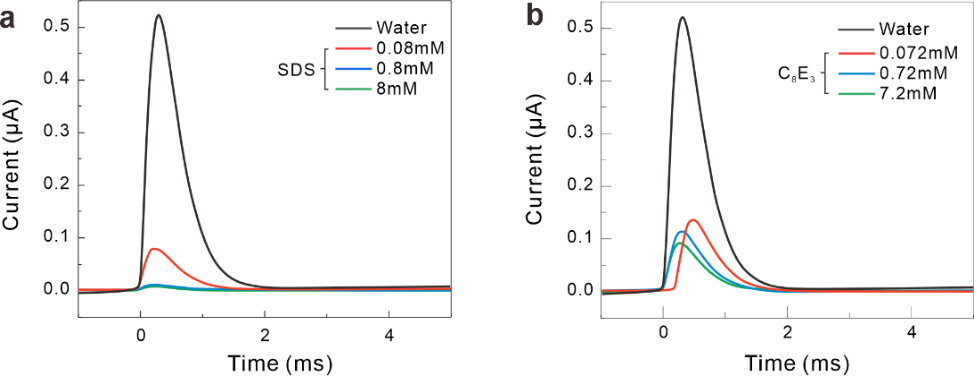


**Figure S1**. Current measured after the drop contacts the electrode after sliding 4 cm. Current traces were recorded for different surfactant concentrations for drops with SDS (a) or C_8_E_3_ (b).


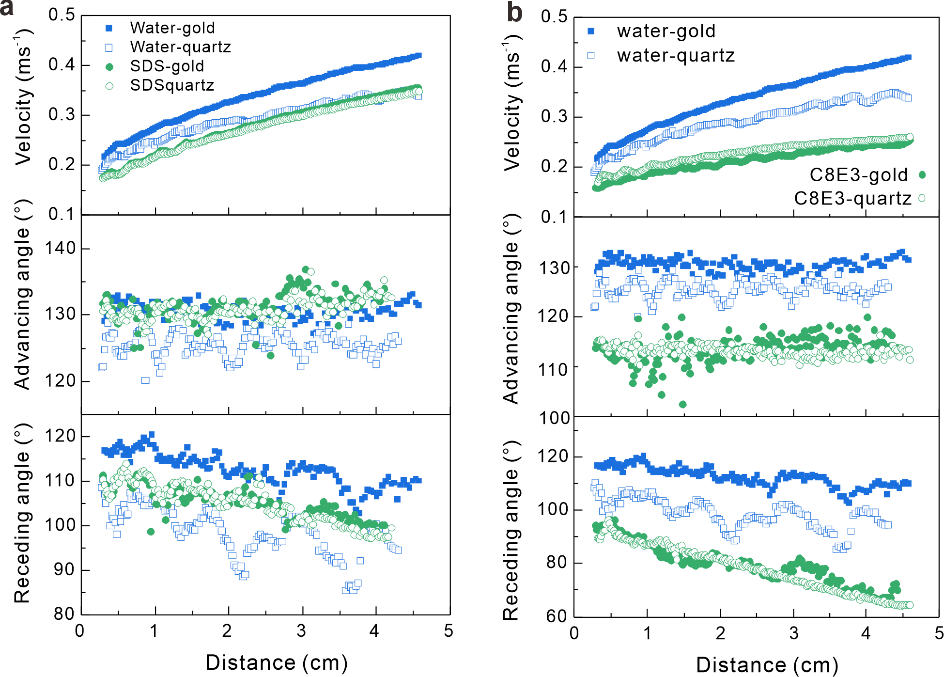


**Figure S2**. Drop velocity, advancing angle and receding angle of sliding drops with (a) 10%CMC SDS and (b) 10% CMC C_8_E_3_ comparing with the case of water drops.


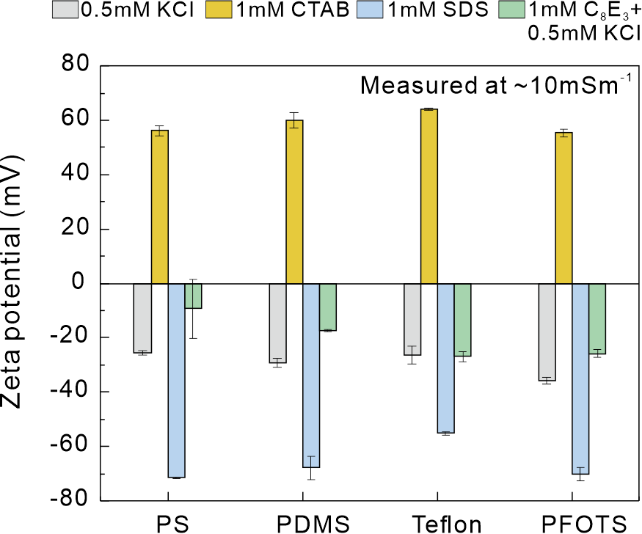


**Figure S3**. Zeta potential of water with 0.5 mM KCl, 1 mM CTAB, 1 mM SDS, and the mixture of 1 mM C_8_E_3_ and 0.5 mM KCl at conductivity of 10 mSm^-1^.


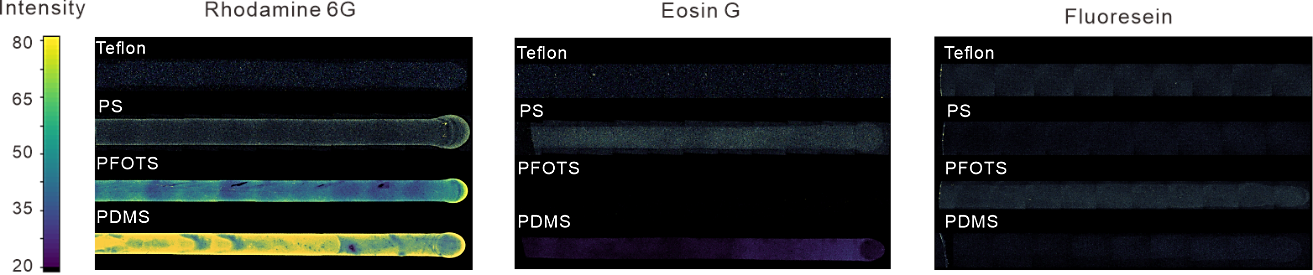


**Figure S4**. Confocal images of drop paths with ~6 cm length and 6 mm width after water drops with fluorescein，eosin G, and rhodamine 6G at a concentration of 0.1mM sliding on fresh Teflon, PS, PFOTS and PDMS coated quartz surfaces.


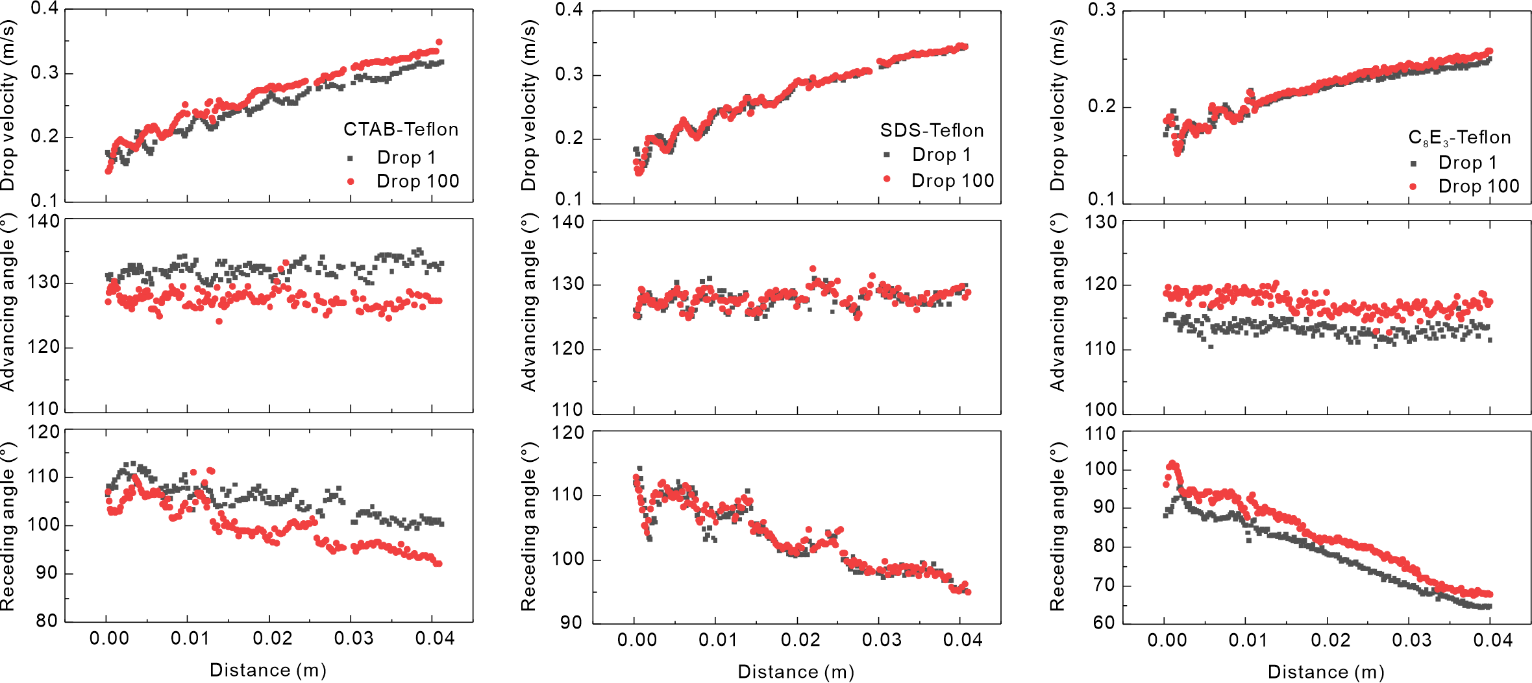


**Figure S5**. Drop velocity, advancing angle and receding angle for of the subsequent 1^st^ (black) and the 100^th^ (red) sliding water drops with 10%CMC CTAB, SDS, and C_8_E_3_ on Teflon-quartz surfaces. The slight decrease in contact angle of the 100th drop compared to the 1st drop for CTAB indicates the deposition of CTAB to the Teflon surface. While the slightly increase in contact angle of the 100th drop compared to the 1st drop for C8E3 indicates that the 1st drops still encounter electrode wetting effect due to less charge reduction by C_8_E_3_.


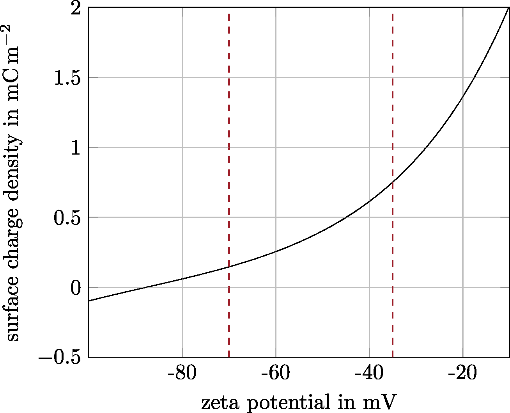


**Figure S6**. Bound surface charge density calculated by equation (S3 and S4) as a function of the effective zeta potential $\zeta$. The equilibrium constants $K_{A}$ and $K_{B}$ are $4.33\cdot{10}^{-4} m^{3}/\mathrm{mol}$ and $3.44\cdot{10}^{-5}\mathrm{mol}/m^{3}$, respectively.


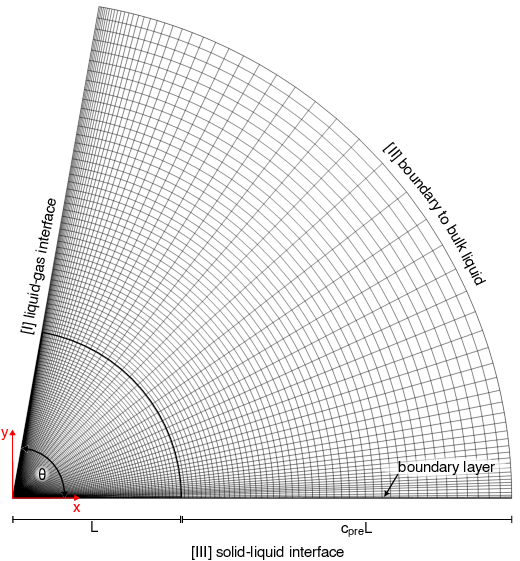


**Figure S7**. Computational domain and schematic grid structure


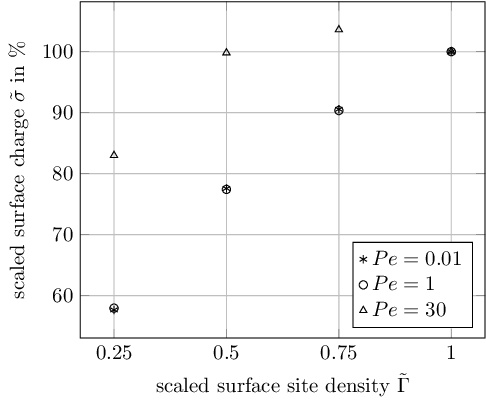


**Figure S8**. Scaled surface charge $\tilde{\sigma}=\sigma/\sigma(\tilde{\Gamma}=1)$ at the contact line at Péclet numbers $Pe$ of 0.01, 1 and 30 over the scaled surface site density $\tilde{\Gamma}=\Gamma/\Gamma_{max}$ with $\Gamma_{max}=55 \mathrm{nm}^{-2}$.
